# Longitudinal immune cell dynamics and heterogeneous trajectories in children with scrub typhus: a descriptive pilot study

**DOI:** 10.1186/s12887-026-06708-7

**Published:** 2026-03-17

**Authors:** Yuanyuan Hua, Yan Zhang, Jimin Zhou, Yan Shi, Feng Xu

**Affiliations:** 1 Department of Pediatrics, the First People’s Hospital of Liangshan Prefecture; Department of Intensive Care Unit Children’s Hospital of Chongqing Medical University, National Clinical Research Center for Children and Adolescents’ Health and Diseases, Ministry of Education Key Laboratory of Child Development and Disorders, Chongqing Key Laboratory of Pediatric Metabolism and Inflammatory Diseases, Xichang, Chongqing People’s Republic of China; 2 Department of Pediatrics, Jin Yang County People’s Hospital, Xichang, People’s Republic of China; 3 Department of Pediatrics, the First People’s Hospital of Liangshan Prefecture, Xichang, People’s Republic of China; 4 Department of Intensive Care Unit Children’s Hospital of Chongqing Medical University, National Clinical Research Center for Children and Adolescents’ Health and Diseases, Ministry of Education Key Laboratory of Child Development and Disorders, Chongqing Key Laboratory of Pediatric Metabolism and Inflammatory Diseases, Chongqing, People’s Republic of China

**Keywords:** Scrub typhus, Children, Immune dynamics, T cells, Monocytes, Natural killer cells

## Abstract

**Background:**

Scrub typhus is an acute febrile illness caused by *Orientia tsutsugamushi*. The dynamic characteristics of immune responses in children with scrub typhus remain unclear.

**Methods:**

Eight children with confirmed scrub typhus were enrolled in this study. Peripheral blood samples were collected sequentially before and after treatment. Absolute counts of B cell, T cell, dendritic cell, monocyte, and natural killer (NK) cell subsets were measured by multicolor flow cytometry. Linear mixed models and ordinary linear regression analyses were performed to evaluate the dynamics of immune parameters with days after fever onset and days of treatment. A *P* value < 0.05 was considered statistically significant.

**Results:**

Linear mixed models showed that the random intercept variances were zero for all immune subsets. Ordinary linear regression analyses revealed that: absolute counts of total T cells decreased significantly with days of treatment (β = -12066.87, 95% CI: -23966.96 to -166.78, *P* = 0.04); absolute counts of CD16 + CD14+ double-positive monocytes increased significantly with days of treatment (β = 130.87, 95% CI: 45.24 to 216.50, *P* = 0.005); absolute counts of CD56^dim^ NK cells increased significantly with days of treatment (β = 1994.06, 95% CI: 337.47 to 3650.65, *P* = 0.021). Days after fever onset showed no significant effect on CD4/CD8 ratio (β = -0.34, 95% CI: -0.79 to 0.12, *P* = 0.144), while days of treatment showed a positive trend with CD4/CD8 ratio (β = 0.59, 95% CI: -0.07 to 1.24, *P* = 0.078). No significant linear associations with days after fever onset or days of treatment were detected for absolute counts of B cell subsets, CD4 + T cells, CD8 + T cells, regulatory T cells, follicular helper T cells, Th1, Th2, Th17, dendritic cells, CD16 + monocytes, total NK cells, CD56^bright^ NK cells, or NKT cells (all *P* > 0.05).

**Conclusion:**

In children with scrub typhus, total T cells decreased with treatment, while CD16 + CD14+ double-positive monocytes and CD56^dim^ NK cells increased with treatment. Most immune subsets showed no significant linear changes associated with disease course or treatment.

**Supplementary Information:**

The online version contains supplementary material available at 10.1186/s12887-026-06708-7.

## Introduction

Scrub typhus is an acute febrile illness caused by *Orientia tsutsugamushi* and transmitted through mites. It poses a significant public health threat in endemic regions of the Asia-Pacific [1–3].The incidence of scrub typhus in Sichuan Province has been increasing annually, mainly distributed in Panzhihua City and Liangshan Yi Autonomous Prefecture. The disease exhibits a seasonal unimodal distribution, with peak incidence in August [4, 5]. The clinical manifestations of scrub typhus are diverse, primarily including fever, rash, eschar, hepatosplenomegaly, and lymphadenopathy. Without timely treatment, the disease can be complicated by pneumonia, meningitis, disseminated intravascular coagulation, and even multiple organ failure [6]. Although early treatment with antibiotics such as doxycycline is effective, the immunopathological mechanisms of the disease, particularly in children, remain inadequately elucidated. Current understanding of the immune response to scrub typhus is mainly derived from animal models and studies in adult patients. Animal model studies have shown that *Orientia tsutsugamushi* infection can induce excessive activation of Th1 responses and suppression of Th2 responses. This immune imbalance is closely associated with endothelial dysfunction and lethality [7, 8]. In adult patients, expansion and activation of circulating NK cells with high IFN-γproduction have been observed [9]. However, longitudinal immunological studies specifically targeting the pediatric population remain scarce.

Based on this, the present study employed a prospective design with sequential sampling during the febrile phase and after defervescence in a cohort of children diagnosed with scrub typhus. Multicolor flow cytometry was used to longitudinally analyze the dynamics of innate immune cells, adaptive immune cells, and regulatory immune cells. The objectives of this study were: (1) to characterize the dynamic changes of immune parameters in relation to disease course and treatment in children with scrub typhus; and (2) to explore the potential associations between immune dynamics and clinical course.

## Method

### Study subjects

A total of eight children with a clinical diagnosis of scrub typhus were enrolled in this study. Patients were included immediately after the clinical diagnosis of scrub typhus was made and the decision to initiate doxycycline or azithromycin therapy was confirmed.

### Diagnostic criteria

According to the 5th Edition of Pediatric Infectious Diseases [10] and Chinese Expert Consensus on the Clinical Diagnosis and Treatment of Scrub Typhus (2024) [11]: the diagnosis of scrub typhus is classified into two levels: clinical diagnosis and laboratory-confirmed diagnosis.

Clinical diagnosis is based on: Epidemiological history (outdoor activities in endemic areas within three weeks before onset); Clinical manifestations (acute fever, eschar/ulcer, lymphadenopathy, rash); Auxiliary laboratory tests (Weil-Felix test with OXK titer ≥ 1:160).

Laboratory-confirmed diagnosis requires, in addition to clinical diagnosis, any of the following: Positive specific IgM antibody; Four-fold or greater rise in specific antibody titers in paired sera; Positive PCR detection of Orientia tsutsugamushi DNA; Isolation of the pathogen.

### Actual inclusion criteria of this study

This study was a descriptive pilot conducted in a resource-limited setting. Patients were enrolled at a primary hospital in Liangshan Prefecture, where the only available serological test for scrub typhus during the study period was the Weil-Felix test (OXK titer ≥ 1:160). Specific Orientia tsutsugamushi IgM antibody testing and PCR were not available at this facility. Therefore, patient inclusion followed the clinical diagnosis criteria defined in the consensus.

#### Ethics statement

This study was approved by the Institutional Review Board of the First People’s Hospital of Liang Shan Prefecture (Approval No: 2023-008). Informed consent was obtained from all participants’ legal guardians.

#### Sample collection

The day of hospital admission, which was also the first sampling time point and prior to treatment initiation, was defined as day of treatment 0. Subsequent blood sampling time points were obtained after treatment with doxycycline or azithromycin. Meanwhile, taking the first day of fever as the starting point (0), the blood sampling time points were defined as days after fever onset and days of treatment based on these two different starting points. Details are shown in Supplementary Table 4.

#### Flow cytometry

Blood samples from the enrolled children were collected via ethylenediaminetetraacetic acid (EDTA) anticoagulant tubes, and the plasma samples used for cytokine measurements were separated and frozen at − 80 ◦C in a freezer for examination. Additionally, peripheral blood mononuclear cells (PBMCs) were immediately isolated via density gradient centrifugation for flow cytometry using human lymphocyte isolation solution. RPMI 1640 medium containing 2% fetal bovine serum was added to freshly extracted PBMCs, which were mixed well, and 200 µl of the cell mixture was added to a 96-well plate such that each well contained 1 × 10^6 cells. Centrifugation (500 ×g, 5 min, 4 ◦C) was performed, the supernatant was removed, and stain was added for surface immunostaining of the PBMCs.

Dead cells were removed with 7AAD dye. The surface labeling antibodies used included those specific to CD3, CD4, CD8, CD25, CD39, CD45, HLA-DR, CD123, CD20, CD16, CD19, CD14, CD56, CD27, CD38, IgD, CD24, CD185, CCR6, CXCR3, CD45RO, and TCR γδ. Intracellular staining for FoxP3 was performed following surface staining. All reagents were obtained from BD Pharmingen, except for the TCRγδ antibody, which was obtained from Beckman. After surface staining was completed, the samples were washed, filtered, and then assayed using a flow analyzer (BD FACSCelesta™ SORP). Stained immune cells were tested via FlowJo software (Tristar, San Carlos, CA). Shown in Supplementary Figure 1, Tables S1 and S2.

#### Statistical analysis

Linear mixed models were used to analyze the dynamic changes in absolute counts of immune cell subsets. The results showed that the random intercept variances were zero for all subsets, indicating no significant baseline heterogeneity among the children. Collinearity diagnostics were further performed, and the variance inflation factors for days after fever onset and days of treatment were both 1.737 (less than 5), indicating no significant collinearity between the two variables, allowing them to be included simultaneously in the regression model. Therefore, ordinary linear regression models were employed in this study, with both days after fever onset and days of treatment included as independent variables. P value < 0.05 was considered statistically significant. Absolute counts were calculated based on 1 × 10⁶ PBMCs. Given the exploratory nature of this study, which aimed to screen for immune parameters potentially associated with disease course, no strict correction for multiple comparisons was applied. However, the possibility of false positives was fully considered during result interpretation, and the main findings will be comprehensively evaluated in the discussion section in conjunction with their biological significance. To identify potential outliers, casewise diagnostics were performed for each regression model, with cases having standardized residuals greater than 3 defined as extreme outliers. All statistical analyses and graphical presentations were performed using SPSS 25.0 and GraphPad Prism 9.5.1 software.

## Results

### Clinical characteristics

A total of 8 children with clinically confirmed scrub typhus were enrolled in this study. The ages ranged from 4 to 14 years (median, 8.5 years), including 3 males (37.5%) and 5 females (62.5%). All cases occurred during the scrub typhus epidemic season in Liangshan Prefecture (August to September). All patients had a clear history of outdoor activity. Fever was the chief complaint in all patients. Characteristic eschars were found in all 8 cases (100%), with distribution in the groin (3 cases), scrotum (2 cases), and one case each in the thigh root, postauricular area, left lower abdomen, and perineum. Two patients had red rashes on the trunk before admission, which had subsided by the time of admission. The remaining 6 patients presented with non-pruritic, blanchable red rashes on the trunk upon physical examination at admission. One patient had postauricular lymphadenopathy, while the remaining 7 patients presented with inguinal lymphadenopathy. One patient showed splenomegaly on physical examination and abdominal ultrasound, while the other 7 patients had no hepatosplenomegaly. The Weil-Felix test was performed at 10 days after fever onset, and all results were negative. Due to limited laboratory conditions at the hospital, specific antibody testing and PCR detection for scrub typhus could not be performed. Routine blood tests showed white blood cell counts ranging from 2.08 to 9.03 × 10⁹/L, with one case below the normal range; hemoglobin levels ranged from 95 to 133 g/L; platelet counts ranged from 94 to 292 × 10⁹/L. C-reactive protein levels were elevated in all cases (8.3–72.09 mg/L). Regarding liver function, alanine aminotransferase levels ranged from 15.41 to 94.45 U/L, and aspartate aminotransferase levels ranged from 18.58 to 185.09 U/L, with mild elevations in some patients. Hyponatremia was observed in 2 cases. Five patients (62.5%) received doxycycline treatment, and 3 patients (37.5%) received azithromycin treatment. Fever resolved within 2 days after treatment initiation in all patients. The length of hospital stay ranged from 6 to 7 days (median, 7 days), and all patients improved and were discharged after treatment.

### Analysis of dynamic changes in immune cell subsets

Through longitudinal monitoring of peripheral blood immune cells before and after treatment in 8 children with scrub typhus, we performed ordinary linear regression analyses on the absolute counts of B cell, T cell, dendritic cell (DC), monocyte, and natural killer (NK) cell subsets. Days after fever onset and days of treatment were included simultaneously as covariates in the models to evaluate the dynamic changes of each immune parameter over time.

### Dynamics of B cell subsets

**Fig. 1:**
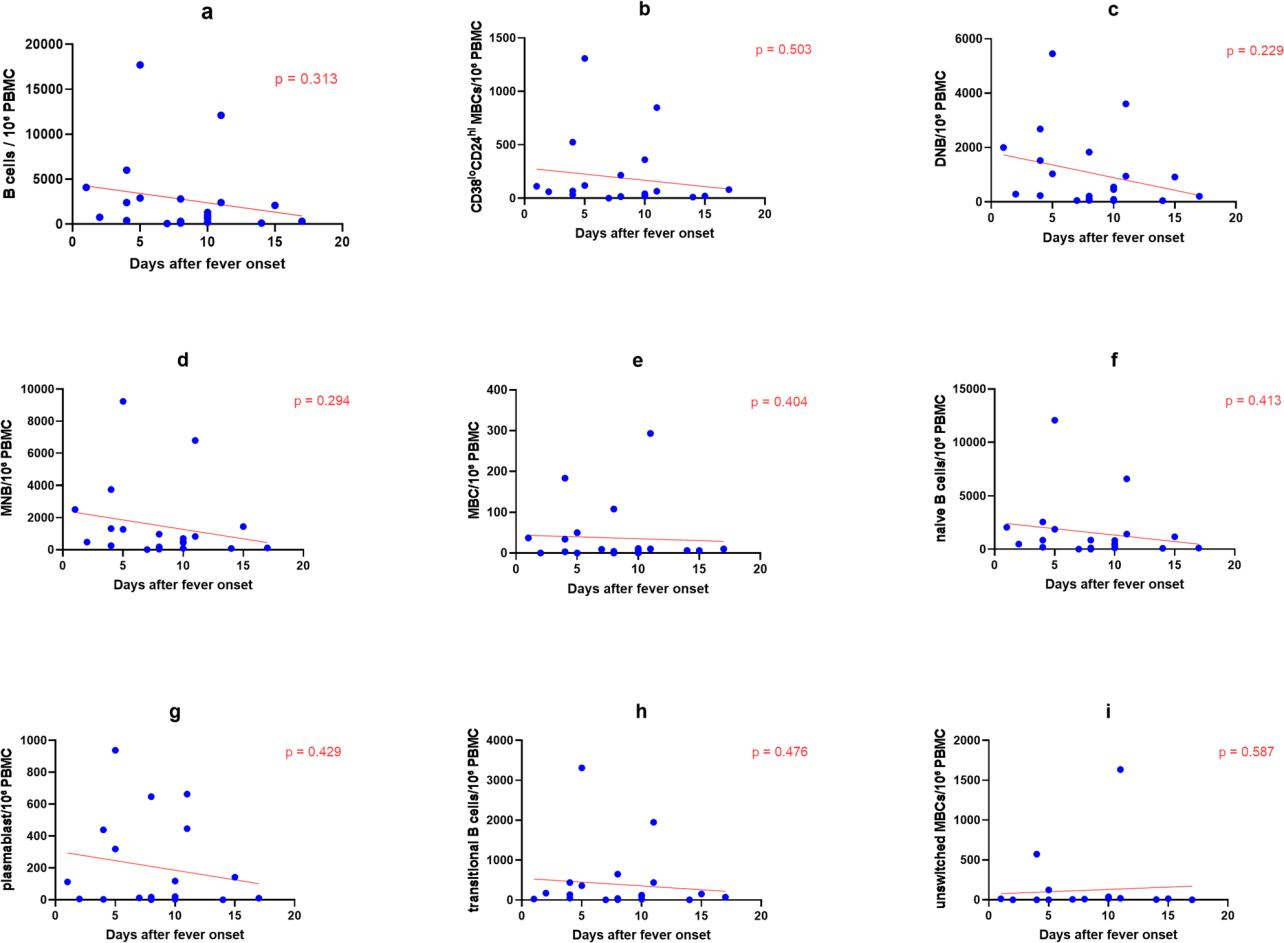
Dynamics of B cell subsets during the course of pediatric scrub typhus. Each dot represents one sample. Red lines indicate linear regression fits adjusted for days of treatment. Absolute cell counts were calculated based on 1×10^6PBMCs and are expressed as cells per 10^6 PBMCs. P values were derived from ordinary linear regression models including days after fever onset and days of treatment as covariates. **a** B cells. **b** CD38loCD24himemory B cells. **c** DNB. **d **MNB,. **e** Memory B cells. **f** Naive B cells. **g** Plasmablasts. **h** Transitional B cells. **i** Unswitched memory B cells

After simultaneously adjusting for days after fever onset and days of treatment, no significant linear trends associated with disease course or treatment were observed in the absolute counts of B cells and their subsets (including plasmablasts, memory B cells, naive B cells, etc.) (*P* > 0.05, Fig. 1, Table 1). These results suggest that, in this study cohort, the dynamic changes of B cells and their subsets may primarily exhibit individual heterogeneity rather than following a uniform population-level linear trajectory.

**Table 1:** Linear regression analysis of B cell subsets including both days after fever onset and days of treatment as covariates

Cell subset	β (95% CI) for days after fever onset	*P*	β (95% CI) for days of treatment	*P*
B cells	-304.49(-917.43,308.44)	0.313	210.06 (-666.32,1086.43)	0.623
CD38^lo^CD24^hi^memory B cells	-15.24(-61.74,31.31)	0.503	7.76( -58.80,74.31)	0.811
DNB	-114.30( -306.09,77.49)	0.229	46.44( -227.78,320.66)	0.728
MNB	-171.48( -502.87,159.92)	0.294	118.31( -355.52,592.14)	0.609
MBC	-4.18( -14.40,6.04)	0.404	7.16( -7.46,21.77)	0.320
Naive B cells	-172.60( -567.42,222.22)	0.413	25,160(-11420,61740)	0.167
Plasmablasts	-15.22(-54.45,24.02)	0.429	6.96(-49.14,63.05)	0.799
Transitional B cells	-39.03(-150.97,79.92)	0.476	43.65( -116.42,203.71)	0.587
Unswitched MBC	-13.44( -64.15,37.27)	0.587	42.44( -30.07,114.95)	0.237

Standardized residual analysis identified Case1 as having extreme elevations in multiple B cell subsets during the acute phase (day 5 after fever onset/day 1 after treatment) (Supplementary Table 6).

### Dynamics of T cell subsets

**Fig. 2:**
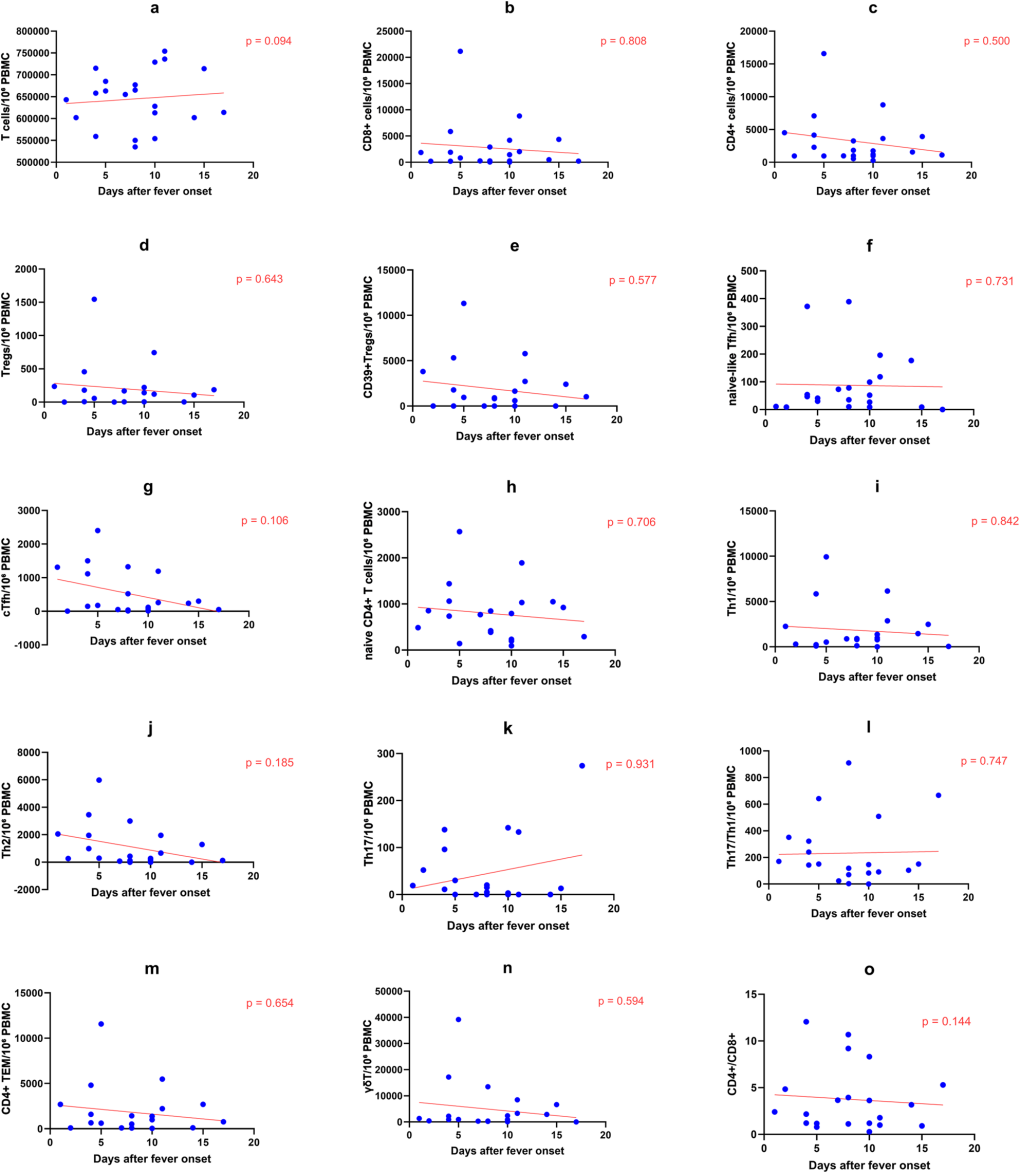
Dynamics of T cell subsets during the course of pediatric scrub typhus. Each dot represents one sample. Red lines indicate linear regression fits adjusted for days of treatment. Absolute cell counts were calculated based on 1×10^6 PBMCs and are expressed as cells per 10^6 PBMCs. P values were derived from ordinary linear regression models including days after fever onset and days of treatment as covariates. **a** T cells. **b** CD8+T cells. **c** CD4+ T cells. **d**Tregs. **e** CD39+Tregs. **f** Naïve-like Tfh cells. **g **cTfh. **h** Naïve CD4+ T cells. **i **Th1. **j **Th2. **k **Th17. **l **Th17/Th1. **m **CD4+ TEM. **n** γδT. **o **CD4/CD8

The absolute counts of total T cells showed a significant decreasing trend with prolonged days of treatment (β = -12066.87, 95% CI: -23966.96 to -166.78, *P* = 0.04), indicating a decline in the overall activation level of T cells after effective treatment. However, after simultaneously adjusting for days after fever onset and days of treatment, no significant linear trends associated with either independent variable were detected in the absolute counts of CD4 + T cells, CD8 + T cells, regulatory T cells (Tregs), follicular helper T cells (Tfh), or functional subsets (Th1, Th2, Th17, etc.) (all *P* > 0.05). Days after fever onset showed no significant effect on the CD4/CD8 ratio (β = -0.34, 95% CI: -0.79 to 0.12, *P* = 0.144), indicating that the ratio did not change linearly with prolonged fever duration. Days of treatment showed a positive trend with the CD4/CD8 ratio, but this did not reach statistical significance (β = 0.59, 95% CI: -0.07 to 1.24, *P* = 0.078), suggesting a potential recovery tendency of the ratio after effective treatment, which requires further validation with an expanded sample size (Fig. 2, Table 2).

**Table 2:** Linear regression analysis of T cell subsets including both days after fever onset and days of treatment as covariates

Cell subset	β (95% CI) for days after fever onset	*P*	β (95% CI) for days of treatment	*P*
T cells	7012.29( -1310.59,15335.16)	0.094	-12066.87( -23966.96,-166.78)	0.04
CD4 + T	-173.73( -0.700.03,352.58)	0.500	-0.27.27( -779.78,725.25)	0.500
Treg	-11.43(-62.06,39.19)	0.643	-0.35( -72.73,72.03)	0.992
CD39 + Tregs	-106.22(-495.69,283.24)	0.577	-37.73( -594.59,519.13)	0.889
CD8 + T	-76.55( -757.30,604.20)	0.817	-103.05( -1076.38,870.29)	0.828
Naive-like Tfh	-2.64( -18.43,13.14)	0.731	4.44( -18.13,27.01)	0.687
cTfh	-71.92( -160.56,16.72)	0.106	25.15( -101.59,151.89)	0.684
CD4 + TEM	-83.28(-464.45,297.90)	0.654	-49.63(-594.63,495.38)	0.852
Th17	-0.40( -9.09,9.89)	0.931	8.82(-4.75,22.39)	0.191
Th17/Th1	-5.56( -40.88,29.77)	0.747	15.24( -35.26,65.75)	0.537
Th1	-34.90(-395.08,325.28)	0.842	-60.53(-575.52,454.45)	0.809
Th2	-133.66( -336.33,69.01)	0.185	8.93(-280.85,298.71)	0.950
Naive CD4 + T cells	-15.81(-101.79,70.17)	0.706	-7.31( -130.25,115.63)	0.903
γδ T	-334.17( -1617.63,949.29)	0.594	-56.67(-1891.77,1778.42)	0.949
CD4/CD8	-0.34(-0.79,0.12)	0.144	0.59(-0.07,1.24)	0.078

Analysis of T cell subsets revealed that Case1 also showed extensive elevations during the acute phase (day 5 after fever onset/day 1 after treatment), affecting CD4 + T cells, CD8 + T cells, regulatory T cells, CD4 + TEM cells, γδ T cells, and NKT cells (Supplementary Table 6).

### Dynamics of dendritic cell and monocyte subsets

**Fig. 3:**
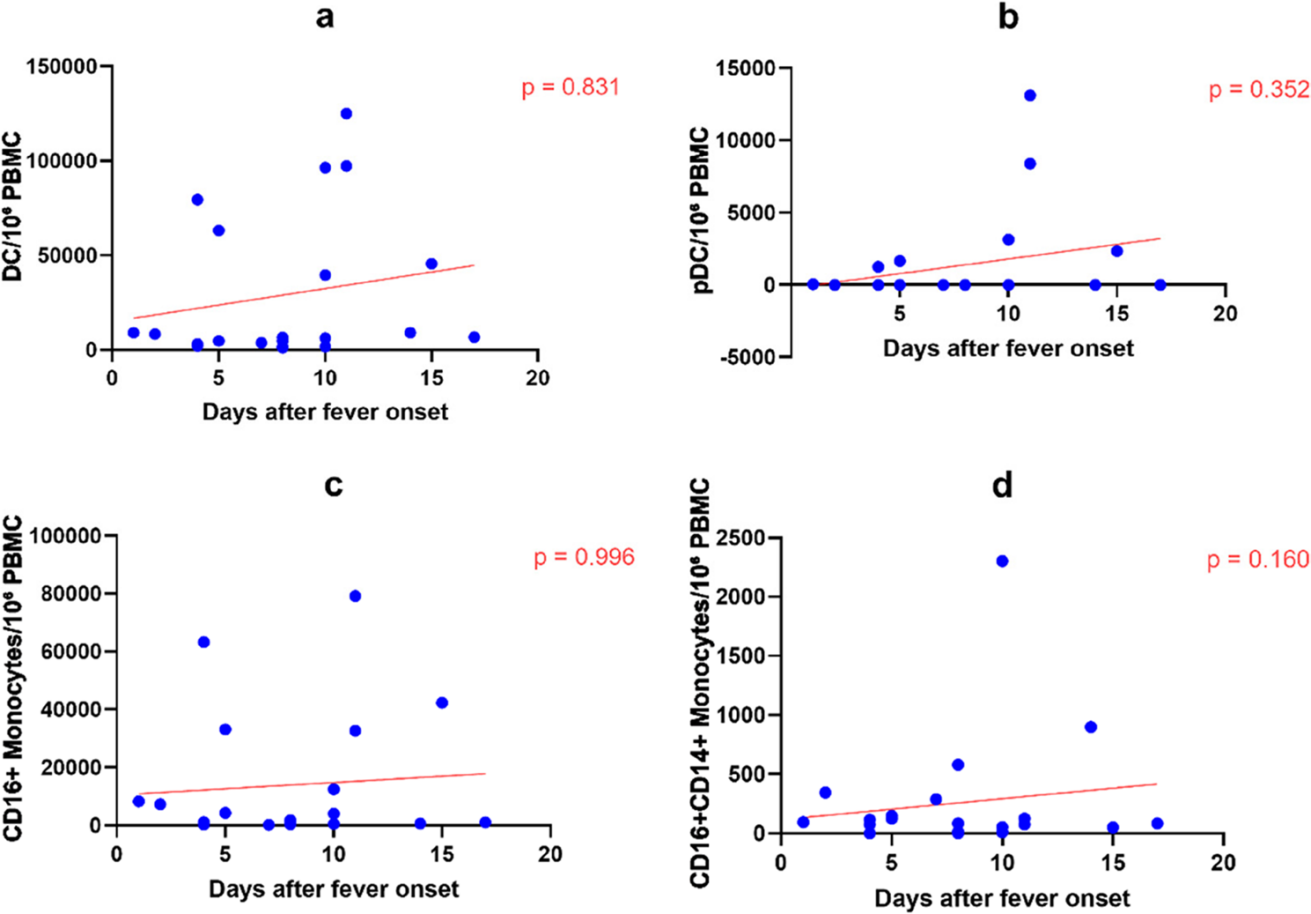
Dynamics of DC cell subsets during the course of pediatric scrub typhus. Each dot represents one sample. Red lines indicate linear regression fits adjusted for days of treatment. Absolute cell counts were calculated based on 1×10^6 PBMCs and are expressed as cells per 10^6 PBMCs. P values were derived from ordinary linear regression models including days after fever onset and days of treatment as covariates. **a** DC. **b** pDC. **c** CD16+Monocytes. **d** CD16+CD14+ Monocytes

After simultaneously adjusting for days after fever onset and days of treatment, no significant linear trends associated with disease course or treatment were observed in the absolute counts of total DCs, plasmacytoid dendritic cells (pDCs), or CD16 + monocytes (*P* > 0.05). Notably, the absolute counts of CD16 + CD14+ double-positive monocytes showed a significant increasing trend with prolonged days of treatment (β = 130.87, 95% CI: 45.24 to 216.50, *P* = 0.005) (Fig. 3, Table 3).

**Table 3:** Linear regression analysis of DC cell subsets including both days after fever onset and days of treatment as covariates

Cell subset	β (95% CI) for days after fever onset	*P*	β (95% CI) for days of treatment	*P*
DC	563.83(-4871.54,0.600)	0.831	2596.01( -5175.52,10367.54)	0.495
pDC	208.86(-247.88,665.01)	0.352	-14.96(-668.02,638.10)	0.962
CD16 + monocyte	7.62( -3221.76,3237.00)	0.996	930.79( -3686.60,5548.18)	0.679
CD16 + CD14+ monocyte	-41.91(-101.80,17.99)	0.160	130.87( 45.24,216.50)	0.005

In dendritic cell and monocyte subsets, Case1 exhibited elevated pDC levels during the convalescent phase (day 11 after fever onset/day 7 of treatment), whereas Patient Case8 showed elevated CD16 + CD14+ double-positive monocytes during the convalescent phase (day 10 after fever onset/day 8 after treatment) (Supplementary Table 6).

### Dynamics of natural killer cell subsets

**Fig. 4:**
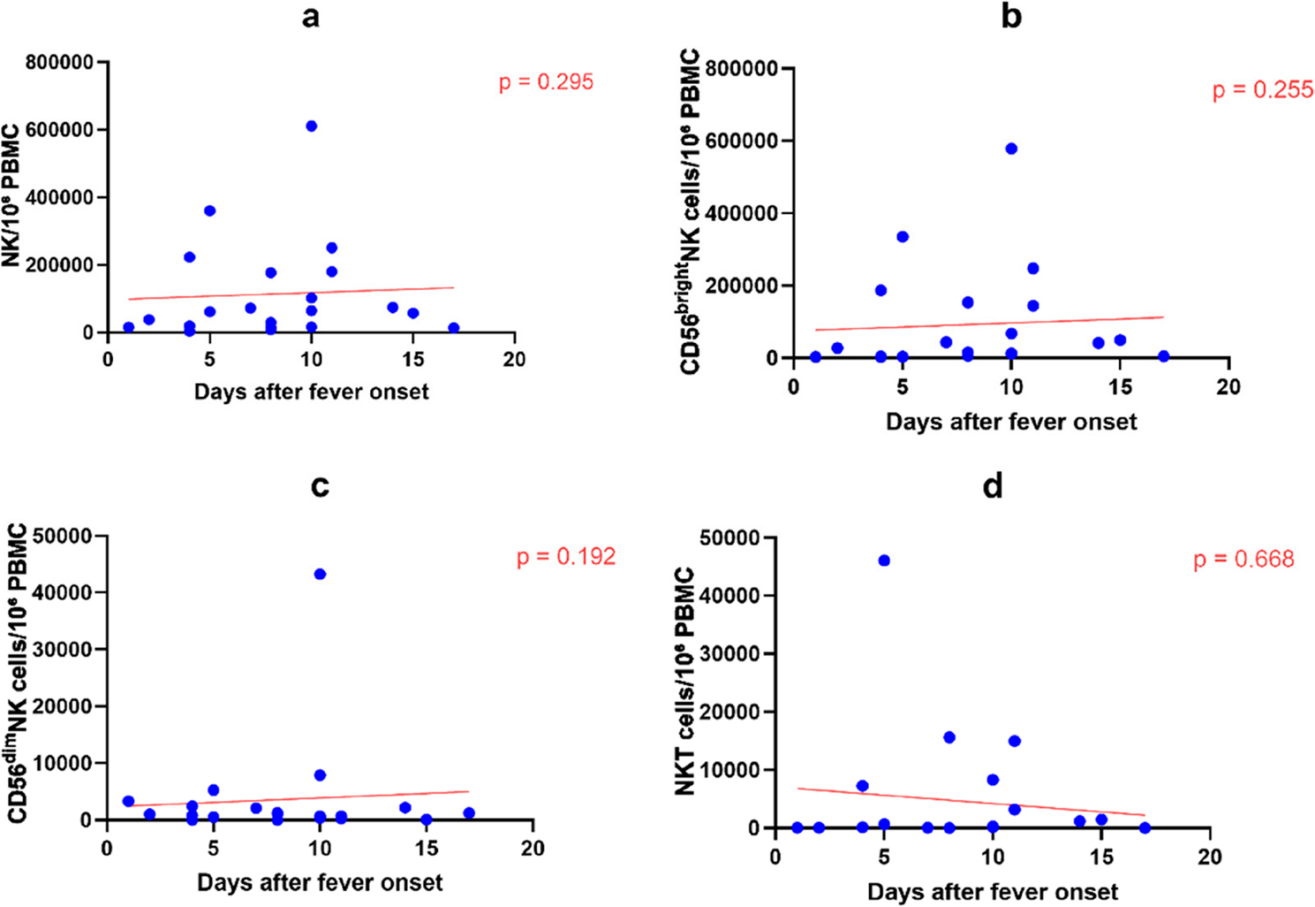
Dynamics of NK cell subsets during the course of pediatric scrub typhus. Each dot represents one sample. Red lines indicate linear regression fits adjusted for days of treatment. Absolute cell counts were calculated based on 1×10^6 PBMCs and are expressed as cells per 10^6 PBMCs. P values were derived from ordinary linear regression models including days after fever onset and days of treatment as covariates. **a** NK. **b** CD56brightNK. **c** CD56dimNK. **d** NKT

After simultaneously adjusting for days after fever onset and days of treatment, no significant linear trends associated with disease course or treatment were observed in the absolute counts of total NK cells, CD56^bright^ NK cells, or NKT cells (all *P* > 0.05). However, the absolute counts of CD56^dim^ NK cells showed a significant increasing trend with prolonged days of treatment (β = 1994.06, 95% CI: 337.47 to 3650.65, *P* = 0.021) (Fig. 4, Table 4).

**Table 4:** Linear regression analysis of NK cell subsets including both days after fever onset and days of treatment as covariates

Cell subset	β (95% CI) for days after fever onset	*P*	β (95% CI) for days of treatment	*P*
Nkcell	10502.97(-9855.13,30861.07)	0.295	-18466.50(-47574.64,10641.64)	0.201
CD56^bright^NK	11064.89(-8600.67,30730.45)	0.255	-19484.12( -47602.06,8633.82)	0.164
CD56^dim^NK	-750.26( -1908.87,408.35)	0.192	1994.06( 337.47,3650.65)	0.021
NKT	-315.03( -1820.01,1189.96)	0.668	63.32(-2088.52,2215.16)	0.952

## Discussion

Through longitudinal monitoring, this study systematically characterized the temporal changes in peripheral blood immune responses in children with scrub typhus. Based on ordinary linear regression analyses, we evaluated the dynamic patterns of each immune parameter in relation to days after fever onset and days of treatment.

This study found that total T cell levels decreased significantly with treatment, consistent with the clinical recovery process, suggesting that the overall activation status of T cells may reflect treatment response. However, no significant linear changes associated with disease course or treatment were detected in various T cell subsets (CD4+, CD8+, Tregs, Tfh, Th1, Th2, Th17, etc.). Several explanations may account for this phenomenon: first, the changes in T cell subsets might exhibit nonlinear characteristics (such as an initial increase during the acute phase followed by a decrease during convalescence), which linear models failed to capture; second, substantial inter-individual heterogeneity may exist, requiring larger sample sizes for further validation; third, changes in T cell subsets in peripheral blood may not fully reflect the immune status in local tissues.Linear regression analysis showed that days after fever onset had no significant effect on the CD4/CD8 ratio (β = -0.34, *P* = 0.144), while days of treatment showed a positive trend with the CD4/CD8 ratio that did not reach statistical significance (β = 0.59, *P* = 0.078). This result suggests a potential recovery tendency of the ratio after effective treatment, which requires further validation with an expanded sample size. Studies in adult patients have observed a decrease in CD4 + T lymphocytes during the acute phase and a relative proliferation of CD8 + T cells during convalescence, leading to an inverted CD4/CD8 ratio [12]. Persistent CD4/CD8 inversion suggests that scrub typhus infection may lead to depletion or functional inhibition of the CD4 + T cell pool [12]. The reduction in CD4 + T cells, particularly Tregs, may disrupt immune homeostasis, leading to dysregulated CD8 + T cell responses and impaired helper function for B cells, collectively affecting the formation of humoral immune memory [12, 13]. Hong et al. demonstrated that the peripheral blood CD4/CD8 ratio has diagnostic value in differentiating scrub typhus from other febrile illnesses [14]. Hauptmann et al. based on mouse model studies, emphasized the dual role of CD8 + T cells in infection control and immunopathology [15]. Inthawong et al. found that during the acute phase of scrub typhus, upon stimulation with pathogen antigens, the main source of cytokines such as IFN-γ and TNF was CD4 + T cells, which exhibited multifunctional characteristics; in contrast, the response of CD8 + T cells was very weak [16].

This study found that the absolute counts of CD16 + CD14+ double-positive monocytes showed a significant increasing trend with prolonged days of treatment (β = 130.87, *P* = 0.005). This double-positive subset may represent an intermediate state of monocytes with specific functions, and their expansion during convalescence suggests a potential role in inflammation resolution, tissue repair, or immunoregulatory processes. Notably, no significant linear trends associated with disease course or treatment were detected in the absolute counts of CD16 + monocytes (*P* > 0.05). Liang et al. demonstrated that in a mouse model of *Orientia tsutsugamushi* infection, TNF-α signaling through the TNFR1 pathway drives the activation and expansion of myeloid cells (including monocytes/macrophages), and TNFR1/2-deficient mice exhibited significant deficiencies in both the total number and activation status of myeloid cells [17]. This mechanistic finding provides a reference for understanding the potential role of monocytes in the immune response to scrub typhus.

Regression analysis revealed that the absolute counts of CD56^dim^ NK cells increased significantly with prolonged days of treatment (β = 1994.06, *P* = 0.021). The CD56^dim^ subset constitutes the predominant population of mature cytotoxic NK cells in peripheral blood, and their expansion following treatment may reflect the restoration or modulation of innate immune function during convalescence. In contrast, no significant linear associations with days after fever onset or days of treatment were observed for absolute counts of total NK cells, CD56^bright^ NK cells, or NKT cells (*P* > 0.05). Kang et al. reported a marked expansion of circulating NK cells accompanied by robust IFN-γ production in adult patients with acute scrub typhus [9]. Additionally, previous studies have demonstrated that while circulating NKT cell numbers remain unchanged during acute scrub typhus, their function is impaired, with functional recovery observed during convalescence [18]. The treatment-associated increase in CD56^dim^ NK cells observed in our study partially aligns with findings in adult populations; however, the absence of significant changes in other NK cell subsets in pediatric patients may reflect age-related differences in immune development.

Mechanistic studies have established that NK cell activation and function during early *Orientia tsutsugamushi* infection are critically dependent on IFN-γ signaling [19]. Furthermore, Manser et al. demonstrated that NK cells in children predominantly express NKG2A, with an age-dependent transition to a KIR-dominated phenotype, which may influence their capacity to respond to infectious challenges [20].

Gonzales et al. demonstrated that in a severe murine model of scrub typhus, infection can lead to disruption of splenic germinal center architecture and downregulation of key signaling pathways involved in B cell activation [21]. Germinal centers are critical sites for the generation of high-affinity memory B cells and long-lived plasma cells [22, 23]. Furthermore, the same research group reported elevated levels of autoantibodies in scrub typhus patients, which may be associated with disease severity [24]. These findings provide mechanistic insights into the complexity of B cell responses in scrub typhus. The absence of significant linear changes in B cell subsets in the peripheral blood of pediatric patients in our study may suggest that B cell responses primarily occur in lymphoid tissues or require longer follow-up to become detectable in the circulation.

In this study, casewise diagnostics (standardized residuals > 3) revealed substantial inter-individual heterogeneity, primarily concentrated in two patients: Case1 and Case8.

Patient Case1 exhibited widespread elevations across multiple immune lineages during the acute phase, including B cells, T cells, NKT cells, and pDCs, with 12 cell subsets showing concurrent increases. Notably, Case1’s clinical data demonstrated markedly elevated CRP and LDH levels, accompanied by liver enzyme abnormalities. This temporal concordance between broad immune activation and elevated inflammatory markers suggests that Case1’s immune response pattern may be consistent with an “acute hyperinflammatory phenotype”. Previous studies have shown that acute scrub typhus is characterized by predominant CD4 + T cell responses with IFN-γ and TNF production [16], and that NK cells are activated and numerically increased during infection [9]. The widespread immune activation observed in Case1 may reflect an enhanced Th1-type response [16], and the elevated LDH and liver enzymes are consistent with tissue damage associated with excessive immune activation reported in severe scrub typhus [7, 8].

In contrast, Case8 showed selective elevations only in CD56^dim^ NK cells and CD16 + CD14+ double-positive monocytes during the convalescent phase (day 10 after fever onset/day 8 after treatment), with clinical inflammatory markers substantially lower than those of Case1. CD56^dim^ NK cells are the predominant effector NK cell subset in peripheral blood which have been shown to be activated and numerically increased during the acute phase of scrub typhus [9]. The expansion of CD16 + CD14+ double-positive monocytes during the convalescent phase may reflect the potential role of this subset in immunomodulation. Previous studies have shown that Orientia tsutsugamushi exhibits tropism for monocytes and dendritic cells during the early stage of infection, with infected monocytes predominantly displaying a CD14 + phenotype and expressing activation markers such as HLA-DR [25]. These findings suggest that monocytes not only serve as target cells for the pathogen but may also participate in antigen presentation and immunoregulation. Furthermore, studies have demonstrated that levels of the monocyte activation marker sCD14 are significantly elevated during the acute phase of scrub typhus and remain above normal levels during convalescence, indicating persistent activation of monocytes following infection [26]. These observations are consistent with the selective expansion of CD16 + CD14+ double-positive monocytes observed in Case8 during the convalescent phase, which may reflect functional activation of this subset during immune reconstitution and its potential role in inflammation resolution.

It should be emphasized that, given the small sample size and the exploratory nature of this study, the observed associations between immune phenotypes and clinical parameters require validation in larger cohorts. The above interpretations should be regarded as reasonable hypotheses based on the available data, aimed at providing preliminary clues for future research.

## Conclusion

This longitudinal study of 8 children with scrub typhus found that total T cells decreased significantly with treatment, while CD16 + CD14+ double-positive monocytes and CD56^dim^ NK cells increased significantly with treatment. Most immune subsets showed no significant linear changes associated with disease course or treatment. No significant baseline heterogeneity was observed across immune subsets. These findings provide preliminary data on immune dynamics in children with scrub typhus.

As a small-sample exploratory study, this investigation has several limitations: first, the small sample size may preclude detection of immune changes with small effect sizes; second, the model did not include random slopes, precluding analysis of inter-individual differences in rates of change; third, only peripheral blood immune cell counts were analyzed, without assessment of cellular function. Fourth, all patients in this study were clinically diagnosed cases rather than laboratory-confirmed cases, which is a key limitation. Due to resource constraints at the enrolling primary hospital, specific Orientia tsutsugamushi IgM testing and PCR were not available. Although all patients tested negative for Weil-Felix antibodies, they all presented with typical clinical manifestations (100% had a characteristic eschar) and defervesced within 48 h after treatment, meeting the criteria for clinical diagnosis. Future studies with larger laboratory-confirmed cohorts are warranted to validate these findings, employ random slope models to analyze inter-individual trajectory differences, and correlate immune phenotypes with clinical parameters.

## Supplementary Information

Supplementary Material 1.

Supplementary Material 2.

## Data Availability

The datasets used and/or analysed during the current study are available from the corresponding author on reasonable request.

## Abbreviations

7AAD: 7-Aminoactinomycin D
ALT: Alanine aminotransferase
AST: Aspartate aminotransferase
CCR6: C-C chemokine receptor 6
CD: Cluster of differentiation
CI: Confidence Interval
CRP: C-reactive protein
CXCR3: C-X-C chemokine receptor 3
CXCR5: C-X-C chemokine receptor 5
DC: Dendritic cell
EDTA: Ethylenediaminetetraacetic acid
FBS: Fetal bovine serum
HLA-DR: Human leukocyte antigen-DR isotype
IFN-γ: Interferon-gamma
IL: Interleukin
KIR: Killer-cell immunoglobulin-like receptors
NK: Natural killer
NKG2A: Natural killer group 2 A
NKT: Natural killer T
O. tsutsugamushi: Orientia tsutsugamushi
PBMC: Peripheral blood mononuclear cell
PCR: Polymerase chain reaction
pDC: Plasmacytoid dendritic cell
TCR: T cell receptor
Tfh: Follicular helper T cell
Th: T helper
TNF-α: Tumor necrosis factor-alpha
TNFR1: Tumor necrosis factor receptor 1
Treg: Regulatory T cell
