# Supplementary material for: Longitudinal immune cell dynamics and heterogeneous trajectories in children with scrub typhus: a descriptive pilot study
Source: BMC Pediatr. 2026 Mar 17;26:370. doi: 10.1186/s12887-026-06708-7 (PMC13107700; doi:10.1186/s12887-026-06708-7)
Supplement: Supplementary file 1 — Supplementary Material 1. [file 12887_2026_6708_MOESM1_ESM.docx]

Supplements

Table 1 Reagents and supplies used in this study

| Reagent / Supply | Brand | Catalog Number |
| --- | --- | --- |
| EDTA blood collection tube | BD Vacutainer® | 367863 |
| Lymphoprep™ | STEMCELL Technologies | 07851 |
| RPMI 1640 medium | Gibco | 11875-093 |
| Fetal bovine serum (FBS) | Gibco | 10099-141 |
| 96-well plate | Corning | 3799 |
| 7AAD viability dye | BD Pharmingen | 559925 |
| FoxP3 staining kit | BD Pharmingen | 560403 |
| Flow cytometry tube | Falcon | 352235 |

Table 2 Antibodies used for flow cytometry

| Cell | Antibody | Fluorophore | Clone | Catalog # | Brand |
| --- | --- | --- | --- | --- | --- |
| T cell | AAD | B690-A | — | 559925 | BD Pharmingen |
|  | CD45 | B525-A | 2D1 | 560367 | BD Pharmingen |
|  | CD3 | R780-A | SK7 | 345763 | BD Pharmingen |
|  | CD8 | V525-A | RPA-T8 | 560662 | BD Pharmingen |
|  | CD4 | V660-A | RPA-T4 | 560650 | BD Pharmingen |
|  | CD127 | R712-A | HIL-7R-M21 | 562661 | BD Pharmingen |
|  | CD25 | V450-A | M-A251 | 560355 | BD Pharmingen |
|  | CD39 | Y610-A | TU66 | 563679 | BD Pharmingen |
| Th cell | CD4 | V660-A | RPA-T4 | 560650 | BD Pharmingen |
|  | CD3 | Y585-A | SK7 | 345763 | BD Pharmingen |
|  | CD185 (CXCR5) | R660-A | RF8B2 | 562471 | BD Pharmingen |
|  | CD45RO | V780-A | UCHL1 | 559865 | BD Pharmingen |
|  | CCR6 | V525-A | 11A9 | 563704 | BD Pharmingen |
|  | CXCR3 | Y780-A | 1C6 | 562558 | BD Pharmingen |
| B cell | CD3 | R780-A | SK7 | 345763 | BD Pharmingen |
|  | CD19 | R660-A | HIB19 | 555415 | BD Pharmingen |
|  | CD27 | V450-A | M-T271 | 560447 | BD Pharmingen |
|  | IgD | B525-A | IA6-2 | 555778 | BD Pharmingen |
|  | CD24 | Y585-A | ML5 | 555428 | BD Pharmingen |
|  | CD38 | B690-A | HIT2 | 555462 | BD Pharmingen |
| NK cell | TCR γδ | R660-A | B1 | 333141 | Beckman Coulter |
|  | CD3 | Y585-A | SK7 | 345763 | BD Pharmingen |
|  | CD56 | V780-A | B159 | 557747 | BD Pharmingen |
|  | CD16 | B690-A | 3G8 | 560470 | BD Pharmingen |
| DC cell | CD14 | V780-A | M5E2 | 560358 | BD Pharmingen |
|  | CD16 | B690-A | 3G8 | 560470 | BD Pharmingen |
|  | CD20 | R860-A | L27 | 340955 | BD Pharmingen |
|  | Lineage (CD3/CD56) | R780-A | SK7/B159 | 345763/557747 | BD Pharmingen |
|  | HLA-DR | B525-A | G46-6 | 560367 | BD Pharmingen |
|  | CD123 | V450-A | 9F5 | 560363 | BD Pharmingen |
| Treg | FoxP3 | Alexa Fluor 488 | 259D | 560403 | BD Pharmingen |

Table 3 Flow cytometry gating strategy for immune cell subsets

| Cell Category | Cell Subset | Definition |
| --- | --- | --- |
| Viability | Live cells | 7AAD⁻ |
|  | Dead cells | 7AAD⁺ (excluded from analysis) |
| B cells | Total B cells | CD19⁺ among lymphocytes |
|  | Naive B cells | IgD⁺CD27⁻ among CD19⁺ cells |
|  | Memory B cells | CD27⁺ among CD19⁺ cells |
|  | Unswitched memory B cells | IgD⁺CD27⁺ among CD19⁺ cells |
|  | Transitional B cells | CD24⁺CD38⁺ among CD19⁺ cells |
|  | Plasmablasts | CD24⁻CD38⁺ among CD19⁺ cells |
|  | DNB cells | IgD⁻CD27⁻ among CD19⁺ cells |
|  | CD38loCD24hi cells | CD38loCD24hi among CD19⁺ cells |
| T cells | Total T cells | CD3⁺ among lymphocytes |
|  | CD4⁺ T cells | CD4⁺ among CD3⁺ cells |
|  | CD8⁺ T cells | CD8⁺ among CD3⁺ cells |
|  | Treg cells | CD25⁺FoxP3⁺ among CD4⁺ T cells (FoxP3 intracellular staining) |
|  | CD39⁺ Tregs | CD39⁺ among Treg cells |
|  | γδ T cells | TCRγδ⁺ among CD3⁺ cells |
|  | Naive CD4⁺ T cells | CD45RO⁻ among CD4⁺ T cells |
|  | CD4⁺ TEM | CD45RO⁺ among CD4⁺ T cells |
| Th cells | Circulating Tfh cells | CXCR5⁺ among CD4⁺ T cells |
|  | Naive-like Tfh | CD45RO⁻CXCR5⁺ among CD4⁺ T cells |
|  | Th1 cells | CXCR3⁺CCR6⁻ among CD4⁺ T cells |
|  | Th2 cells | CXCR3⁻CCR6⁻ among CD4⁺ T cells |
|  | Th17 cells | CXCR3⁻CCR6⁺ among CD4⁺ T cells |
|  | Th17/Th1 double positive | CXCR3⁺CCR6⁺ among CD4⁺ T cells |
| NK cells | NK cells | CD3⁻CD56⁺ among lymphocytes |
|  | CD56^bright^ NK cells | CD56⁺⁺ among NK cells |
|  | CD56^dim^ NK cells | CD56⁺ among NK cells |
|  | NKT cells | CD3⁺CD56⁺ among lymphocytes |
| DC cells | Lineage-negative cells | CD3⁻CD14⁻CD16⁻CD20⁻CD56⁻ among live cells |
|  | Total DCs | HLA-DR⁺ among lineage-negative cells |
|  | pDCs | CD123⁺ among total DCs |
|  | CD16⁺ DCs | CD16⁺ among total DCs |
|  | CD16⁺CD14⁺ cells | CD16⁺CD14⁺ among total DCs |

Table 4 Clinical data for each individual case

|  |  | Case1 | Case2 | Case3 | Case4 | Case5 | Case6 | Case7 | Case8 |
| --- | --- | --- | --- | --- | --- | --- | --- | --- | --- |
| **Demographics** | | | | |  | | | | |
| Sex |  | Female | Male | Male | Female | Female | Female | Female | Male |
| Month of onset |  | August | August | August | September | August | September | August | August |
| Age (years) |  | 4 | 8 | 11 | 14 | 6 | 6 | 9 | 13 |
| **Clinical Course** | | | | |  | | | | |
| Eschar/ulcer  Rash Lymphadenopathy |  | Yes  Yes  Yes | Yes  Yes  Yes | Yes  Yes  Yes | Yes  Yes  Yes | Yes  Yes  Yes | Yes  Yes  Ye | Yes  Yes  Yes | Yes  Yes  Yes |
| FeverDuration |  | 6 | 3 | 12 | 9 | 6 | 6 | 12 | 4 |
| Fever Days at Admission |  | 4 | 1 | 10 | 7 | 4 | 4 | 10 | 2 |
| **Key Laboratory Findings at Admission** | | | | |  | | | | |
| WBC(10^9/L) 5.44 8.04 9.03 | | | | | 4.42 5.68 7.89 6.37 2.08 | | | | |
|  | | | | |  | | | | |
| CRP(mg/L) |  | 62.94 | 70 | 58.9 | 8.3 | 16.17 | 72.09 | 18.5 | 30.7 |
| PLT(10⁹/L) |  | 101 | 265 | 280 | 225 | 265 | 94 | 292 | 171 |
| ALT (U/L) |  | 94.45 | 29.21 | 62.29 | 28.1 | 15.41 | 17.68 | 94.15 | 34.04 |
| AST (U/L) |  | 185.09 | 47.33 | 69.26 | 26.89 | 27.66 | 18.58 | 106.92 | 44.06 |
| Albumin (g/L) |  | 37.91 | 41.56 | 35.98 | 37.68 | 32.99 | 41.56 | 34.79 | 43 |
| LDH(mg/L) |  | 749.4 | 308.7 | 485.9 | 275.9 | 452.7 | 271.5 | 630.2 | 280.4 |
| **Blood Sampling Time Points**  **(day after onset of fever/day of treatment)** | | | | |  | | | | |
| Time Point 1 |  | 4/0 | 1/0 | 10/0 | 7/0 | 4/0 | 4/0 | 10/0 | 2/0 |
| Time Point 2 |  | 5/1 | 5/4 | 17/7 | 8/1 | 8/4 | 8/4 | 11/1 | 8/6 |
| Time Point 3 |  | 11/7 |  |  | 10/3 |  |  | 15/5 | 10/8 |
| Time Point 4 |  |  |  |  | 14/7 |  |  |  |  |

Demographic information, key features of the clinical course, and selected laboratory findings at the time of hospital admission are shown. The timing of peripheral blood sampling for immunological analysis is expressed as days relative to day after onset of fever and day of treatment.

Table 5 Summary of clinical manifestations and Weil-Felix (OXK) test evidence for each case.

| Patient | Fever | Eschar/ulcer | Rash | Lymphadenopathy | Splenomegaly | Treatment Response | Weil-Felix (OXK)Titer |
| --- | --- | --- | --- | --- | --- | --- | --- |
| Case1 | Yes | Yes | Yes | groin | No | Defervescence within 48h | Negative |
| Case2 | Yes | Yes | Yes | groin | No | Defervescence within 48h | Negative |
| Case3 | Yes | Yes | Yes | groin | No | Defervescence within 48h | Negative |
| Case4 | Yes | Yes | Yes | groin | Yes | Defervescence within 48h | Negative |
| Case5 | Yes | Yes | Yes | groin | No | Defervescence within 48h | Negative |
| Case6 | Yes | Yes | Yes | groin | No | Defervescence within 48h | Negative |
| Case7 | Yes | Yes | Yes | groin | No | Defervescence within 48h | Negative |
| Case8 | Yes | Yes | Yes | retroauricular | No | Defervescence within 48h | Negative |

Table 6. Patients with standardized residuals > 3 and their distribution across immune cell subsets

| Patient | Immune cell | days after  fever onset | Days after treatment | standardized residuals |
| --- | --- | --- | --- | --- |
| Case 1 | B cell | 5 | 1 | 3.166 |
| Case 1 | CD38^lo^CD24^hi^memory B cell | 5 | 1 | 3.153 |
| Case 1 | MNB | 5 | 1 | 3.029 |
| Case 1 | MBC | 11 | 7 | 3.146 |
| Case 1 | Naïve B cell | 5 | 1 | 3.491 |
| Case 1 | Transition B cell | 5 | 1 | 3.475 |
| Case 1 | Unswitch B cell | 11 | 7 | 3.662 |
| Case 1 | CD4+T | 5 | 1 | 3.284 |
| Case 1 | Tregs | 5 | 1 | 3.500 |
| Case 1 | CD39+ Treg | 5 | 1 | 3.147 |
| Case 1 | CD8+T | 5 | 1 | 3.576 |
| Case 1 | CD4+TEM | 5 | 1 | 3.340 |
| Case 1 | γδ T | 5 | 1 | 3.495 |
| Case 8 | CD56^dim^ NK | 10 | 8 | 3.567 |
| Case 1 | NKT | 5 | 1 | 3.637 |
| Case 1 | pDC | 11 | 7 | 3.311 |
| Case 8 | CD16+CD14+ monocyte | 10 | 8 | 3.238 |

Standardized residuals > 3 were defined as extreme outliers;this table only includes outliers with standardized residuals > 3 and does not list all immune cell subsets analyzed.
