# Supplementary material for: Longitudinal immune cell dynamics and heterogeneous trajectories in children with scrub typhus: a descriptive pilot study
Source: BMC Pediatr. 2026 Mar 17;26:370. doi: 10.1186/s12887-026-06708-7 (PMC13107700; doi:10.1186/s12887-026-06708-7)
Supplement: Supplementary file 2 — Supplementary Material 2. [file 12887_2026_6708_MOESM2_ESM.pdf]

(a)

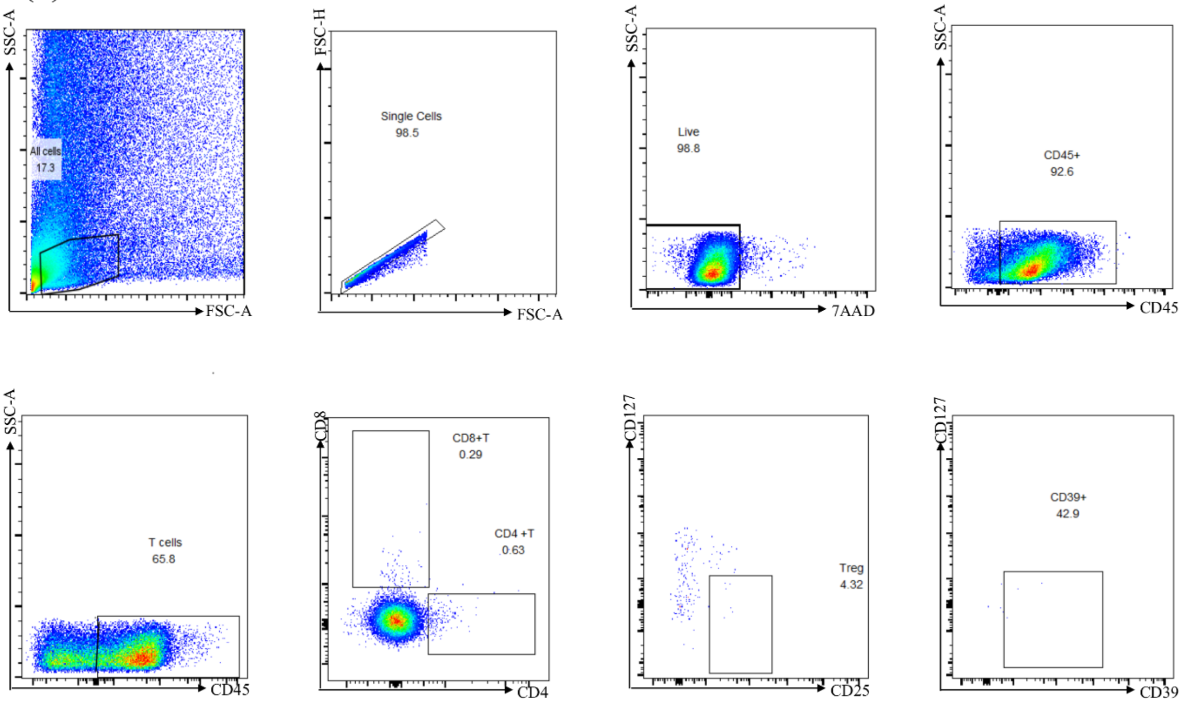
SSC-A
FSC-A
All cells
17.3
FSC-H
FSC-A
Single Cells
98.5
SSC-A
7AAD
Live
98.8
SSC-A
CD45
CD45+
92.6
SSC-A
CD45
T cells
65.8
CD8
CD4
CD8+T
0.29
CD4 +T
0.63
CD127
CD25
Treg
4.32
CD127
CD39
CD39+
42.9

(b)

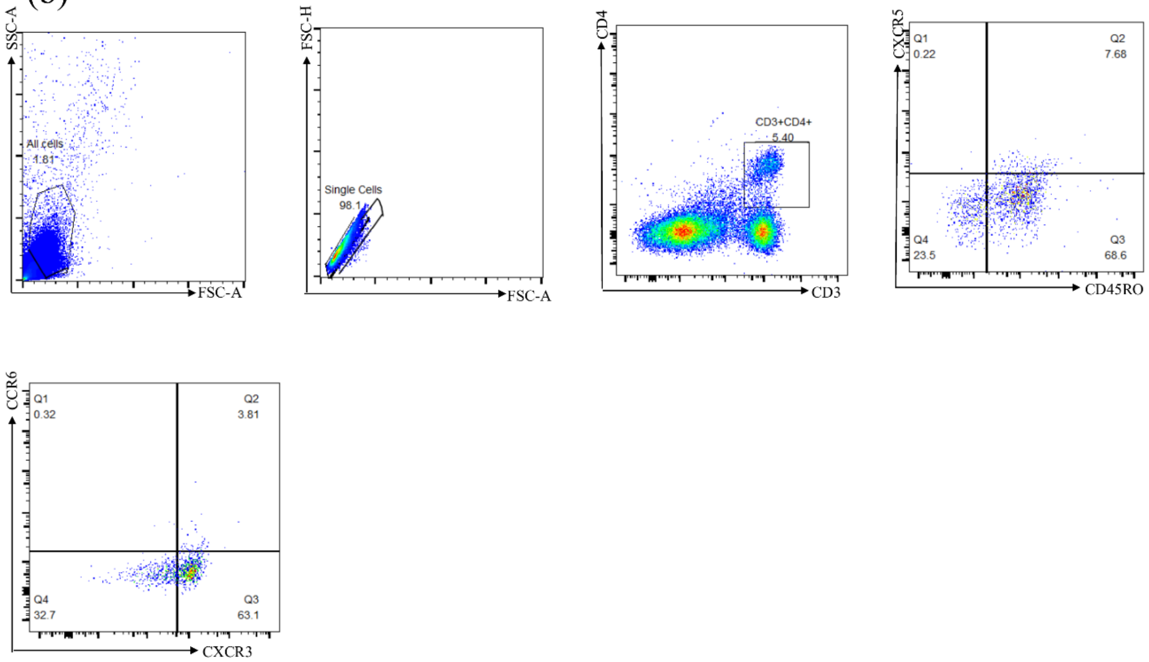
SSC-A
FSC-A
All cells
1.81
FSC-H
FSC-A
Single Cells
98.1
CD4
CD3
CD3+CD4+
5.40
CXCR5
CD45RO
Q1
0.22
Q2
7.68
Q4
23.5
Q3
68.6
CCR6
CXCR3
Q1
0.32
Q2
3.81
Q4
32.7
Q3
63.1

(c)

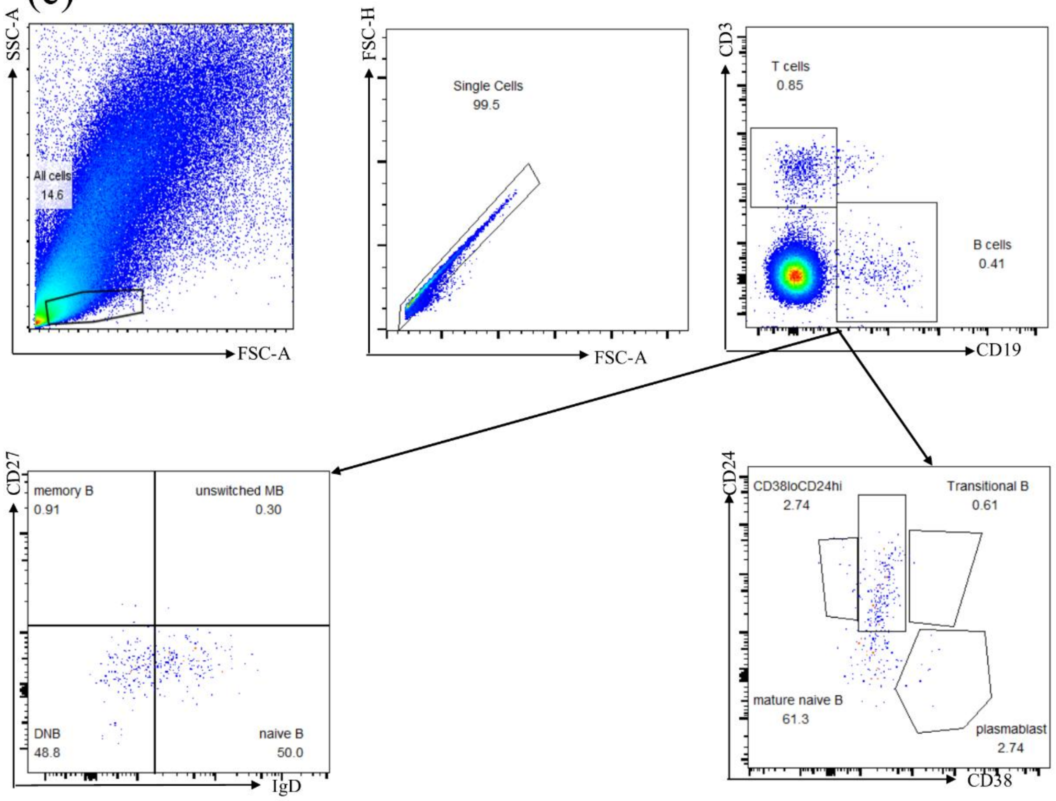
SSC-A
FSC-A
All cells
14.6
FSC-H
FSC-A
Single Cells
99.5
CD3
CD19
T cells
0.85
B cells
0.41
CD27
IgD
memory B
0.91
unswitched MB
0.30
DNB
48.8
naive B
50.0
CD24
CD38
CD38loCD24hi
2.74
Transitional B
0.61
mature naive B
61.3
plasmablast
2.74

(d)

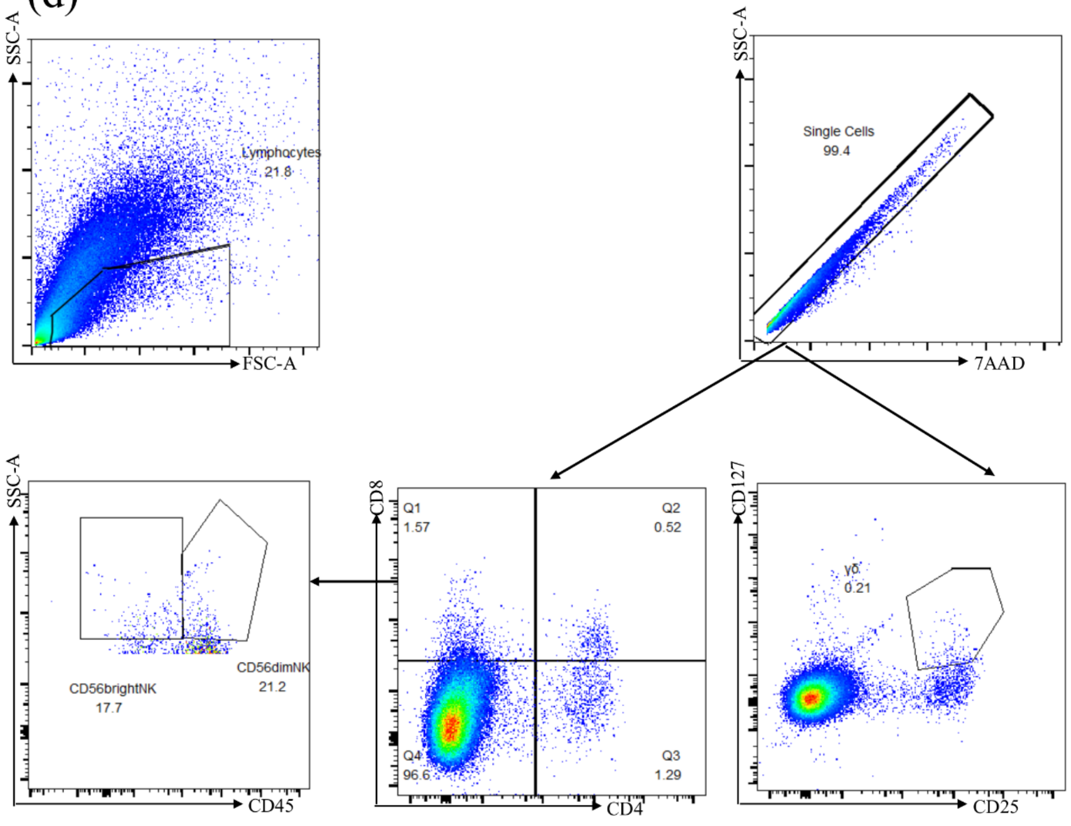
SSC-A
FSC-A
Lymphocytes
21.8
SSC-A
7AAD
Single Cells
99.4
SSC-A
CD45
CD56brightNK
17.7
CD56dimNK
21.2
CD8
CD4
Q1
1.57
Q2
0.52
Q4
96.6
Q3
1.29
CD127
CD25
γδ
0.21

(e)

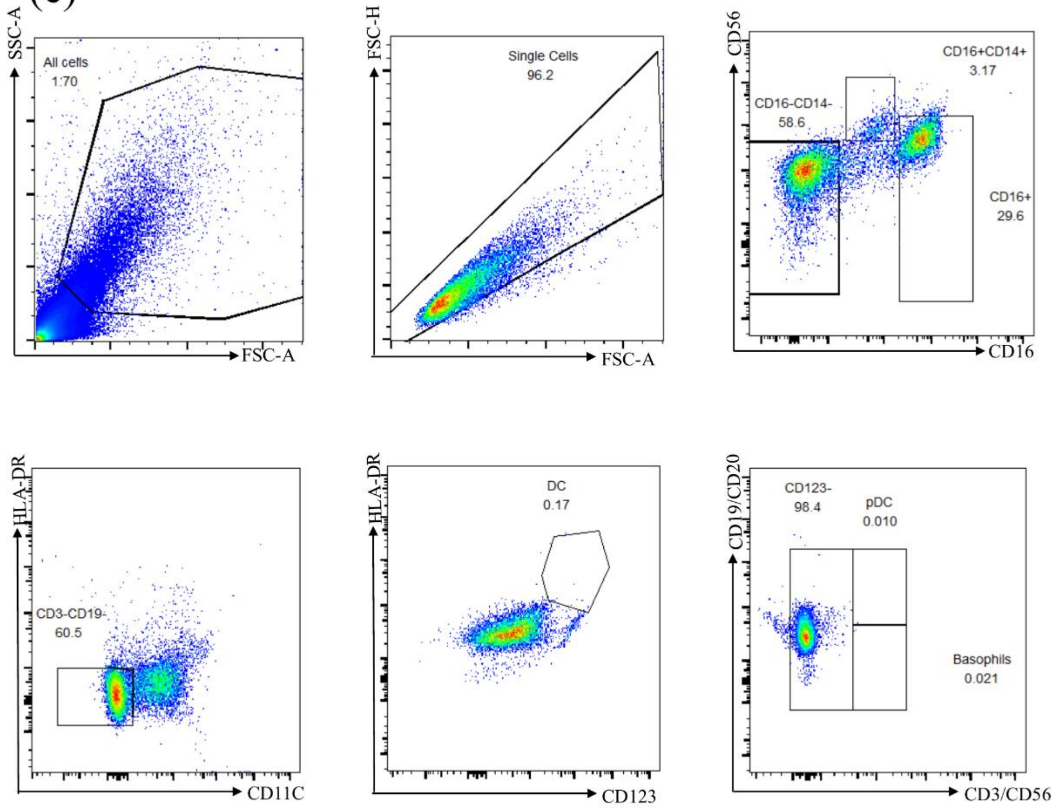

Figure1 Flow loop gate strategy. **(a)** T cells and subset gating strategies. **(b)** T-helper (Th) cells and subsets. **(c)** B cells and subsets. **(d)** Natural killer (NK) cells and subsets. **(e)** Dendritic cells (DCs) and subsets.
